# Printing Technologies as an Emerging Approach in Gas Sensors: Survey of Literature

**DOI:** 10.3390/s22093473

**Published:** 2022-05-03

**Authors:** Nikolay P. Simonenko, Nikita A. Fisenko, Fedor S. Fedorov, Tatiana L. Simonenko, Artem S. Mokrushin, Elizaveta P. Simonenko, Ghenadii Korotcenkov, Victor V. Sysoev, Vladimir G. Sevastyanov, Nikolay T. Kuznetsov

**Affiliations:** 1 Kurnakov Institute of General and Inorganic Chemistry, Russian Academy of Sciences, 31 Leninsky pr., 119991 Moscow, Russia; fisenkonk@yandex.ru (N.A.F.); egorova.offver@gmail.com (T.L.S.); artyom.nano@gmail.com (A.S.M.); ep_simonenko@mail.ru (E.P.S.); vg_sevastyanov@mail.ru (V.G.S.); ntkuz@igic.ras.ru (N.T.K.); 2 Higher Chemical College of the Russian Academy of Sciences, D. Mendeleev University of Chemical Technology of Russia, 9 Miusskaya sq., 125047 Moscow, Russia; 3 Laboratory of Nanomaterials, Skolkovo Institute of Science and Technology, 3 Nobel Str., 121205 Moscow, Russia; f.fedorov@skoltech.ru; 4 Department of Theoretical Physics, Moldova State University, 2009 Chisinau, Moldova; ghkoro@yahoo.com; 5 Department of Physics, Yuri Gagarin State Technical University of Saratov, 77 Polytechnicheskaya Str., 410054 Saratov, Russia

**Keywords:** gas sensor, additive manufacturing, deposition, film, coating, ink, lab-on-chip, multisensor array

## Abstract

Herein, we review printing technologies which are commonly approbated at recent time in the course of fabricating gas sensors and multisensor arrays, mainly of chemiresistive type. The most important characteristics of the receptor materials, which need to be addressed in order to achieve a high efficiency of chemisensor devices, are considered. The printing technologies are comparatively analyzed with regard to, (i) the rheological properties of the employed inks representing both reagent solutions or organometallic precursors and disperse systems, (ii) the printing speed and resolution, and (iii) the thickness of the formed coatings to highlight benefits and drawbacks of the methods. Particular attention is given to protocols suitable for manufacturing single miniature devices with unique characteristics under a large-scale production of gas sensors where the receptor materials could be rather quickly tuned to modify their geometry and morphology. We address the most convenient approaches to the rapid printing single-crystal multisensor arrays at lab-on-chip paradigm with sufficiently high resolution, employing receptor layers with various chemical composition which could replace in nearest future the single-sensor units for advancing a selectivity.

## 1. Introduction

The studies of semiconducting solids in the 1950s [1] resulted in the appearance of the microelectronic industry [2] which has revolutionized our capabilities in working out and sensing the environment. As a part of this mainstream, the pioneer R&D works performed in the 1960s by Sejyama for chemiresistors [3], Baker for thermocatalytic pellistors [4], Lundstrom for gas-sensitive field-effect transistors [5], King and Wohltjen for gas-sensitive bulk and surface acoustic wave detectors [6,7], if to mention major pioneer ones, have allowed us to obtain a number of electronic units capable to “sense” (adapted from Latin “sentire”) the gases and gas mixtures on a real-time scale. Since that time, a number of research labs and companies have extensively expanded the field so that now we have plenty of commercial gas detectors which help us in many industries and ordinary life [8], especially while considering the revolution of the Internet of Things (IoT) concept. Nowadays, there is a solid trend to make the gas-sensing units more powerful in terms of (i) sensitivity, i.e., to have a low detection limit close to ppb range of concentration, (ii) selectivity, i.e., to be able to distinguish the analytes of interest, (iii) low energy consumption, i.e., to ideally work with the help of normal environmental support at “room temperature”, (iv) low response/recovery time, i.e., to react in seconds or faster upon an analyte’s appearance, and (v) stability, i.e., to be able to work normally in the long term without an additional re-calibration. These issues are significantly addressed via proper choosing of an appropriate material [9], as well as the technology [10] to fabricate the units from it.

Historically speaking, three types of material coatings, as sintered powders, and thick and thin films, are employed to develop a commercial gas-sensing unit. So, there have been a number of techniques elaborated to integrate the materials into the sensor, which can be divided into several groups: evaporation- and sputtering-based methods, aerosol-based methods, sol-gel-based methods, and thick-film deposition methods [11]. Among the techniques, the most requested are ones which are able to deposit thin-film layers at a low cost but with a high accuracy. Therefore, the design of such methods and protocols remains to be a major focus of many research groups. Although there are well-established and low-cost methods to deposit semiconductor layers, such as spin coating, rod coating, spray coating, dip coating, chemical vapor deposition (CVD), physical vapor deposition (PVD), etc. [12,13], their primary limitations often relate to (i) a precision of control of the geometry of a deposited layer, (ii) strict requirements to the deposition conditions, for example, existence of vacuum or inert atmosphere, and (iii) general limitations of solid-phase methods because the precursor composition is sometimes difficult to reproducibly adjust.

**Figure 1:**
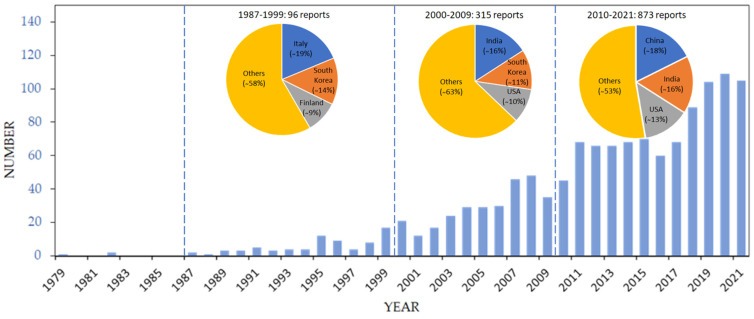
The number of documents published in the printed gas sensor field in 1979–2021 from sources indexed by Scopus (search on 12/04/2022) under the key words of “gas sensor” and “printing”. Insets: the top three countries in the field at approximately decade time periods and their relative portion of publications.

Therefore, a new deposition group of methods has unsurprisingly evolved to meet the requests for controlled deposition of thin-film layers accounting for the existing trend to a miniaturization of functional sensing elements. These methods consider printing [14,15,16,17]. Currently, we observe a series of academic studies to evaluate the properties of the printed layers for gas sensors depending on the applied precursor, equipment, and deposition protocols [13,18,19,20,21]. As shown in Figure 1, the primary publications dealing with employing printed layers in gas sensors date back to 1980–90s [22], though most reports start appearing mainly at the start of the 21st century with a tremendous growth from approximately 10 per year to currently, approximately 100 per year.

It is worth noting that during the 20th century, the leading countries were European (Italy and Finland) together with South Korea, but now, the leading countries are China, India, and the United States, at least in a quantity of reports which seems to be reflecting an investment of these countries to R&D works devoted to low-cost production of gas sensors as well as a general growth of the microelectronic industry in their countries.

Obviously, the major focus of R&D in printed layers for gas sensors lie in thin films because they ordinarily yield improved mechanical and functional performance when compared to thick-film ones. For example, an increase of the layer thickness usually tends to lower the sensitivity of semiconductor material, though, in porous layers, one might observe another trend, due to enhancing the active specific surface [13,23]. The deposition of layers with defined geometry, thickness, and high resolution has been performed and studied using several printing methods, including ink-jet printing, screen printing, gravure printing, microextrusion, and aerosol printing [15,24,25,26]. This emerging field has been observed in various reviews, addressing methods for printing gas sensors on flexible substrates [27,28,29], and the most common contact and non-contact printing methods with an emphasis on ink requirements [30], in some cases, focusing on a particular group of methods [31]. One of the reviews discusses [32] a number of less common approaches. However, new methods of gas sensor component formation have emerged so far to be considered; their impact in solving such urgent problems as designing efficient gas sensors and multisensor arrays should be taken into account under the lab-on-chip paradigm.

In this contribution, we consider printing protocols to deposit semiconducting layers as gas (chemical) sensors and multisensor arrays. Further, we discuss characteristics of layers which are relevant to these applications. The text is organized to cover the following major points:

(1) general overview and classification of printing methods (contact, non-contact, roll-to-roll);
(2) description of characteristics of semiconductor gas sensors and multisensor arrays to be adjusted via fabrication protocols;
(3) features of each printing method while applying to gas sensor fabrication;
(4) characteristics of functional inks employed as the receptor components to design gas (multi)sensors in frames of various printing approaches.

Finally, we summarize the up-to-date literature data in a table to highlight a deposition rate, a resolution, layer thickness, the requirements to ink viscosity, as well as strong and weak points of each printing method, and compositions to design receptor layers for gas detection.

## 2. Methods for Printing Functional Coatings on Various Substrates

Conventionally, printing methods are primarily distinguished by the applied pressure to the substrate, i.e., contact or non-contact ones. The major types of contact methods are: (1) gravure printing; (2) gravure offset printing; (3) flexographic printing; (4) microcontact printing (µCP)); (5) nano-imprinting lithography (NIL)); (6) reverse-offset printing; (7) dip-pen pen nanolithography (DPN); (8) fountain pen printing (FPN); (9) pen plotter printing; (10) microplotter printing; (11) screen printing. The list of non-contact methods includes: (1) ink-jet printing; (2) aerosol jet printing; (3) microextrusion printing; (4) slot-die printing; (5) 3D printing; (6) laser-induced forward transfer (LIFT); (7) dielectrophoretic printing (DEP). The recent popular roll-to-roll method [33] allocates from these two main classes to be a rather complementary one for depositing layers fast and on different substrates. Consequently, the roll-to-roll approach is often incremented in technological processes in the industry.

## 3. Description of Characteristics of Semiconductor Gas Sensors and Multisensor Arrays

One of the major characteristics of a gas sensor is its sensitivity. This parameter matures by several factors which can be formally separated into two groups [30]. The first group includes microstructural properties—shape, size of particles, and porosity of the material [34]. For instance, the shape of crystallites defines the crystallographic planes involved in the gas-sensitive effect. This means that by changing the shape of crystallites, we can influence the catalytic activity of the surface and thus achieve the required sensitivity and selectivity of the sensor response. Furthermore, it is assumed that the smaller the size of crystallites in the gas-sensitive material, the better its gas permeability, i.e., porosity, the greater the sensor response we achieve. In dense layers, the interaction with analytes of interest takes place almost exclusively on the surface, while in the porous layer, it occurs in the bulk of gas-sensitive material. The second group envelops characteristics of the chemical composition of the functional layer—the type of doping, stoichiometry, composition of sensing layer, etc. [35]. This approach suggests that the sensitivity of sensors is controlled not only by the morphology of the receptor gas-sensitive layers but also by the chemical composition of the surface which affects the concentration and type of surface defects as well as the catalytic activity of the surface [36]. All these parameters are usually defined by receptor, transduction, and utility functions, as originally described by Yamazoe [37]. This means that the technology employed to manufacture the sensors must be able to deposit gas-sensitive layers in the necessary composition with the required surface properties. In other words, the composition of the ink utilized in the printing process should be selected in such a way that, on the one hand, it promotes the formation of a porous gas-sensing matrix, and, on the other hand, it does not contain components that reduce a surface activity. The process to form a gas-sensitive layer should also consider a minimum number of post-printing treatments to supply the applied material with the properties of interest.

To build multisensor arrays, gas sensors with different gas-sensing characteristics are ordinarily required, which is achieved by varying the layer thickness, particle size, and composition of the deposited semiconductor layer [38]. As a rule, the solution of this problem goes via taking a number of discrete sensors manufactured in frames of various technologies [39]. In order to reach the higher gas discrimination, the number of fabricated sensors in the array is usually rather high; for example, E. Baldwin et al. [40] employed 26 different sensors for this purpose. As a result, the measuring gas-sensor unit appears to be quite large which makes applying these devices rather difficult in currently developed miniaturized techniques. It is possible to solve this problem only if there are technologies which allow us to design the entire number sensors over the smallest possible area [41]. This means that the manufacturing technology must provide the possibility to reduce sensors to the given size, frequently at submillimeter or micrometer range. Reducing power consumption through the use of micro hotplates and integrating gas sensors with silicon electronics also requires a reduction in sensor size. The optimal method should also provide the possibility of varying the parameters of the formed layer during the printing process. For example, be able to apply simultaneously or sequentially the materials of different composition.

We must also not forget that one of the key conditions for the printed material, ordinarily of semiconducting nature in gas sensors, is the capability to conduct an electric current. The surface morphology often plays a very important role, i.e., cracking/local damage of a layer, uneven thickness, and heterogeneity, just as in the case of the dip-coating method, might limit their applications, e.g., due to the formation of an irreproducible conductance network or the localization of current flow in thicker regions [25]. Thus, we believe that the major characteristics to meet when choosing the method of print layers for gas sensors are: (1) resolution (µm); (2) layer thickness (µm); (3) speed of printing (m/min); (4) ink viscosity (mPa·s); (5) coating uniformity, and (6) other specific features of the method. Some parameters, such as resolution and ink viscosity, are responsible for the minimum size of sensors that can be manufactured using a particular printing method. The coating uniformity contributes greatly to better reproducibility of sensor parameters. The layer thickness can determine the preferred field of application of the sensors being manufactured. It was found that thin films with a thickness of 100 nm are frequently employed in the detection of oxidizing gases such as ozone, while thick films with a thickness of tens of micrometers are preferred in the detection of reducing gases such as CO and H_2_ [42]. The speed of printing is also important because a low printing speed, even yielding excellent sensor parameters, is not suitable for large-scale application due to low productivity.

Thus, it is important to analyze and to pay attention to the change in the electrophysical characteristics of materials in dependence on the printing conditions including the characteristics of the precursor (viscosity, surface tension, concentration, etc.), since the optimization of the printing process will allow the (i) efficient use of the existing printers, and (ii) further improve the printing characteristics in order to develop more precise printing methods. Further, we examine the major methods to print planar-type receptor materials utilized in course of fabricating gas sensors and multisensor arrays.

## 4. Printing Methods to Fabricate Receptor Layers of Gas Sensors

While designing the gas-sensor receptor materials, a great variety of printing technologies are used these days which vary both in the principle of operation and in such parameters as printing speed, spatial resolution, thickness of the formed coatings, and their microstructure, etc. We have summarized the data in Table A1 (Appendix A). The most common approaches in this context are: ink-jet printing, aerosol jet printing, 3D printing, microextrusion printing, pen plotter printing, microplotter printing, screen printing, gravure printing, flexographic printing, laser-induced forward transfer (LIFT), dip-pen nanolithography (DPN), nano-imprinting lithography (NI), and microcontact printing (µCP).

### 4.1. Ink-Jet Printing

**Figure 2:**
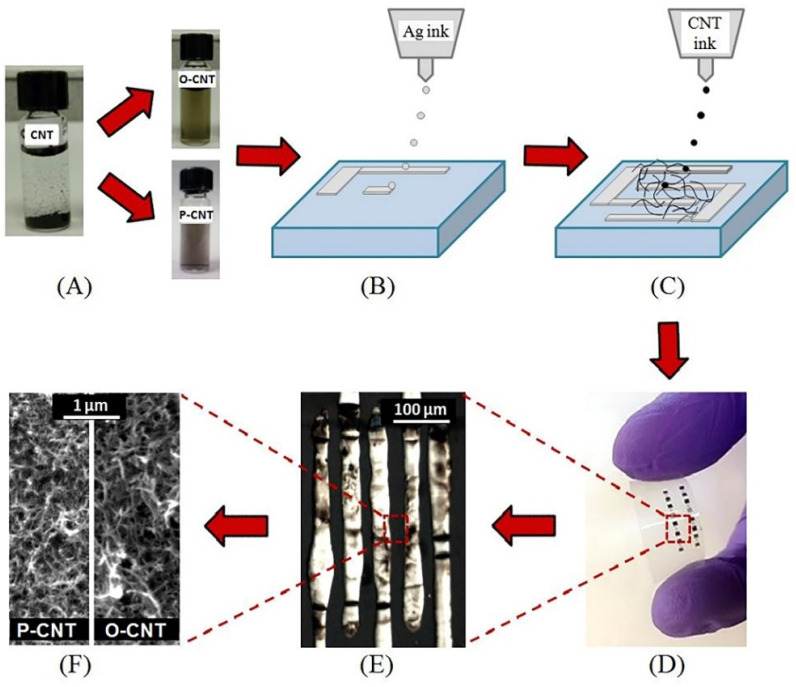
Fully printed and flexible CNT-based gas sensor: (**A**) CNTs functionalization with carboxylic acid (O-CNTs) and PEDOT:PSS (P-CNTs); (**B**) printing of Ag electrodes; (**C**) printing of CNT; (**D**) photograph of the sensor on flexible substrate; (**E**) optical microscope image showing the printed silver interdigitated electrodes, and (**F**) SEM image showing the printed CNTs. Reproduced from ref. [55]. Copyright 2016, Elsevier, Ltd.

Ink-jet printing is a non-contact method for manufacturing many functional layers. It enables a programmable setting of the trajectory to follow for a functional ink on the substrate surface with the ability for rapid changes in the pattern unlike printing methods employing stamps or stencils. The ink-jet printing unit, i.e., printer, includes a cartridge (container) with ink, a printhead, and a control system for the position of the printhead (drives, step motors, etc.). Usually, small droplets, normally at a volume of ca. several picoliters, are jetted from the printhead based on various mechanisms, such as gravity, hydrostatic pressure, as well as piezoelectric, via applying electric and thermal actuators [43]. Ink-jet printing can be classified at two basic categories, continuous ink-jet printing (CIJ) and drop-on-demand (DOD). In the case of the CIJ approach, the liquid flow is generated by the application of hydrostatic pressure followed by transformation of the flow into the droplets under the action of surface tension. To design the required pattern, unwanted droplets are deflected by an electric field to be collected in a gutter. The DOD approach of droplet formation is realized with the help of the thermal, piezoelectric, and electrohydrodynamic protocols. The method generates separate drops to be applied precisely to defined places at the substrate. The parameters of the printed layers primarily depend on the characteristics of the printing unit, of functional inks and substrates [44,45]. One of the major problems associated with ink-jet printing is the possible appearance of so-called “coffee spots” which, when dried, lead to the formation of an inhomogeneous layer. One way to minimize the effect of the “coffee spot” is to maintain a high temperature of the substrate so that the solvent can quickly evaporate prior to undergoing a hydrodynamic spreading of the surface [46,47]. Another solution is the addition of minor amounts of solvents with a high boiling point and a low surface tension to slow evaporation at the contact angle [48]. This technology seems to be quite promising to design electrochemical sensors [49,50,51], photodetectors, electronics [49,51,52,53], and alternative energy applications [50,54]. The advantages usually include a compatibility with biological materials, low cost, programmed control, relatively high spatial resolution down to 20–50 µm, relatively high speed, and performance. Still, this technology has a low printing speed due to the limited number of nozzles, therefore, possibility of clogging the nozzles with solid particles make the scalability of this technology not yet feasible. Ink-jet printing is the most popular method for the machine designing gas-sensor layers due to the widespread use of corresponding devices; it is enough to note household printers which are widely spread out. The composition of the manufactured coatings can vary greatly from noble metals to metal oxides, carbon nanostructures, etc.. As can be seen from Figure 2, Alshammari et al. [55], could form Ag electrodes and a functional layer of polymer-modified carbon nanotubes over flexible substrates with a sufficiently high resolution by using dispersion-type inks.

The prepared sensor demonstrated quite good performance in forward to ethanol detection. Ink-jet printing is also quite effective to apply while employing inks to appear as solutions of necessary reagents or organometallic precursors. For example, nanosized thin films of TiO_2_–10%ZrO_2_ composition developed using alcohol solutions of titanium and zirconium alkoxoacetylacetonates as inks were found to have a high reproducible sensor response to O_2_ [56].

### 4.2. Aerosol Jet Printing

**Figure 3:**
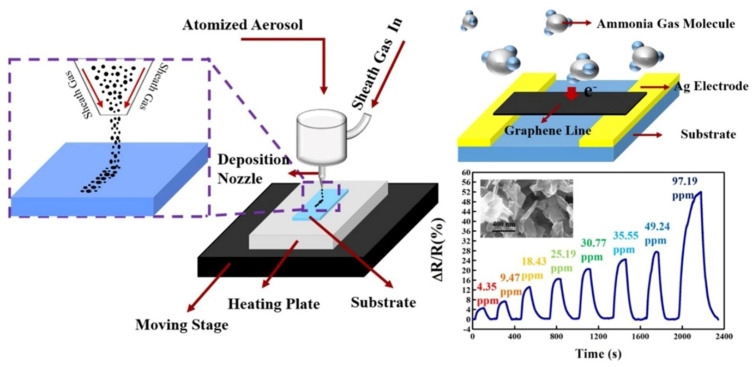
Schematic diagrams of aerosol jet printing to fabricate ammonia gas sensor; dynamic and quantitative responses of the sensor to NH_3_ at various concentrations. Reproduced from ref. [58]. Copyright 2021, Elsevier, Ltd.

Aerosol printing is a direct non-contact method of fabrication of layers on various substrates including ones with curved surfaces. Aerosol printing enables one to reach the resolution of about 20 microns [57]. The mechanism of particle transfer is the following: Primarily, the functional ink is vaporized, after that, a carrier gas is injected capturing the ink vapors, then this mixture passes under pressure through the print head along with the sheath gas, usually N_2_, which prevents the mixture from interacting with the walls of the print head. The mixture is sprayed onto the substrate through a nozzle (Figure 3) [58]. A branched technique, aerosol lithography, has been recently developed by W. Jung et al. [59], who demonstrated 3D printing of flexible metallic nanostructures with the size of elements around 100 nm. Various spirals, lettering, rings, columns, and hanging structures were printed on the Si/SiO_2_ wafer. The gas flow rate through the shell (SHGFR), the flow rates of carrier gas (CGFR), the temperature of the substrate [60], and the printing speed were shown to influence the printing [61]. In the case of aerosol printing, nozzles are resistant to clogging unlike ink-jet printing systems. At the same time, the viscosity range for functional inks employed in frames of this technique is usually much greater, from 1 cP to 1000 cP for a pneumatic sprayer, and from 1 cP to 10 cP for an ultrasound sprayer, which allows one to use a larger range of inks and to more finely adjust the characteristics of the ink, and, so, the layer parameters [62]. One of the actual problems of aerosol printing is an excessive spraying, i.e., overspray (OS) [63], which results in inaccuracies during the printing. In particular, the relationship between OS, SHGFR, and CGFR has attracted reasonable attention.

Aerosol jet printing can be used to produce transparent electrodes for flexible electronics [64], carbon nanotubes [65], and photodetectors [66]. Due to a high productivity, rather high resolution of the formed objects, a large number of degrees of freedom for spatial moving of the printing nozzles, resistance of nozzles to clogging, as well as a wide range of acceptable viscosity of the ink, this method is one of the most effective when designing gas sensors based on receptor materials of different chemical composition. In particular, the work of [58] considers the application of this technology in using a high-sensitive ammonia sensor with the response to be ca. 4.64% to 4.35 ppm and ca. 52.01% to 97.19 ppm of NH_3_. In this case, graphene was utilized as the receptor material, which was applied to the substrate surface by spraying an aqueous dispersion of graphene to form an aerosol. Arsenov et al. [67] showed that aerosol printing can also be effectively used in the fabrication of metal electrodes which are components of miniature gas sensors. For this purpose, dispersed systems based on Pt particles with an average size of about 100 nm, to be sprayed with a pneumatic atomizer, were employed as inks. The technique is also used for the development of gas sensors containing metal oxide layers as receptor components. In a recent work [68], it was reported that the denser, as compared to screen printing, thick-film structures formed by aerosol printing in the Y_2_O_3_-ZrO_2_ system exhibit lower noise and high sensitivity to NO and NO_2_. Therefore, this method is highly versatile and, in dependence on the chemical composition of the receptor material, layer thickness, and printing conditions, makes it possible to fabricate chemical sensors sensitive to various analytes [58,69,70].

### 4.3. 3D Printing

3D printing always requires building a 3D model of an object and converting it to the STL format, which carries only information about the geometry of the surfaces of the three-dimensional model. Then, this file is processed using “slicers”, programs that “slice” the model (for example, Simplify3D, Cura, etc.), and data on the technical parameters of printing, as nozzle movement speed, nozzle temperature, table temperature, etc. After installing the entire set of parameters, the slicer outputs a G-code file written by a software language with a numerical programmed control [71]. The width of the lines and the resolution of 3D printing depends primarily on the diameter of the nozzle, on the characteristics of stepper motors installed in the printer, on the temperature of the nozzle and the table, as well as on the executable G-code.

**Figure 4:**
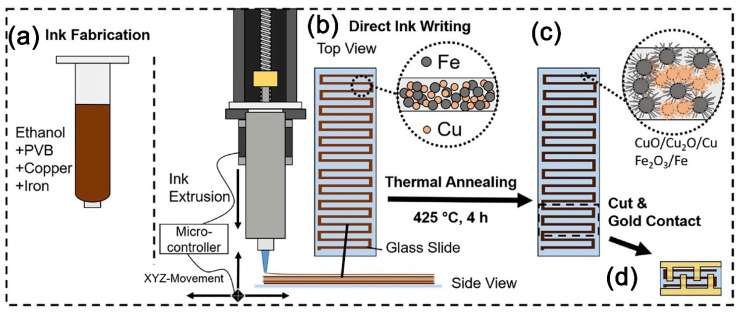
Schematic sensor fabrication process by 3D printing: (**a**) ink fabricated by mixing Cu and Fe microparticles in ethanol, stirring in PVB until a homogeneous state, then the ink is filled into the printer cartridge; (**b**) direct ink writing via piston-driven syringe pumps in a 3D-printing setup; layer by layer building of meandering Cu-Fe stripes; (**c**) The glass slide with the printed object is placed in an oven at air at 425 °C for 4 h where the metal oxide nanostructures are formed; (**d**) Single sensor devices are coated with Au and then cut for further electrical and sensor investigations. Reproduced from ref. [74]. Copyright 2020, Elsevier, Ltd.

Several different protocols have been developed to implement the 3D printing. In addition to stereolithographic printing (SLA), there are the fused deposition modeling (FDM) or fused filament fabrication method (FFF), “ink-jet” 3D printing (or, binder jetting, BJ), selective laser-sintering method (SLS), selective laser melting (SLM), electron beam melting (EBM), by light processing (digital light processing, DLP), and laminated object manufacturing (LOM). Additionally, 3D printing is frequently distinguished to classify into two groups as nozzle-based printing (or nozzle-based 3D printing), which includes, for example, functional ink printing, and light-based 3D writing which includes, for example, two-photon lithography and projection micro-stereolithography [72]. Since the printing protocols which involve a photopolymerization make it possible to achieve a high printing resolution, the extensive work is actively underway to create new materials—photoinitiators [73]. In general, 3D printing, is possibly the most popular method to manufacture gas sensors, similar to ink-jet printing. In particular, this method was effectively used to fabricate a composite sensing material based on Cu and Fe microparticles whose surface was subject to a partial oxidation as a result of additional heat treatment in air [74] (Figure 4). The coating fabricated by this protocol has been characterized as rather selective to acetone vapors to yield a 50% chemiresistive response to 100 ppm of the analyte. The 3D printing, under fused deposition modeling (FDM) protocol, was recently employed to design an NH_3_ gas sensor operated at room temperature [75]. In the first stage of the study, a composite based on metallic Cu particles and polylactic acid (PLA) was made, which was further subjected to a high-temperature treatment to remove the organic component and to oxidize the copper in order to obtain a monoclinic CuO phase. The appeared framework structure of the material has ensured the sensor to have advanced sorption properties and, consequently, a high stability, sensitivity, and selectivity for ammonia detection at room temperature. The 3D printing has also employed other functional layers in electrochemical detectors [75], biosensors [76], photoelectrodes [77,78], photoresist printing [79], biomaterials, porous materials [80,81], flexible electronics [82], optoelectronics [83], production of drugs [84], and perovskite layers [85].

### 4.4. Microextrusion Printing

The microextrusion printers are very similar to conventional 3D ones and consist of a heating element, a slide print bed, step motors that allow moving along the X, Y, Z axes, and ink/paste supply system. In the microextrusion process, ink is squeezed out through the nozzle (or nozzles) pneumatically or mechanically using a piston or a screw. Unlike ink-jet printing, microextrusion applies micro-balls on the surface of the substrate. There are commercially available bioprinters utilizing this method, for example, Inkredible+ (CELLINK, Sweden), which is employed for biomedical applications [86].

The factors to define characteristics of the printed layers include the nozzle size, starting from 0.5 µm to correspond best for liquid ink printing up to 1540 µm when using pastes, ink viscosity in the 1–1 × 10^8^ mPa·s range, and printing speed, from 1 µm/s to 500 µm/s depending on ink type and printing task [87]. This technique has been shown to print gas sensors [88], biological tissues [89,90,91], transparent flexible force sensors employed in wearable sensors under water and in robotics [92], graphene supercapacitors on flexible substrates [93], solid-oxide fuel cells [94,95,96], solar cells [97], electrochemical capacitors [98], thin films and membranes [99]. It is worth noting that the microextrusion printing is still rarely used in the fabrication of inorganic materials [95,100] that limits its application. However, it has great prospects, particularly to form structures at thick-film architecture. In this way, microextrusion printing can be compared to screen printing in terms of its efficiency, although there is no need to prefabricate templates that correspond to the geometrical parameters of the target coatings.

**Figure 5:**
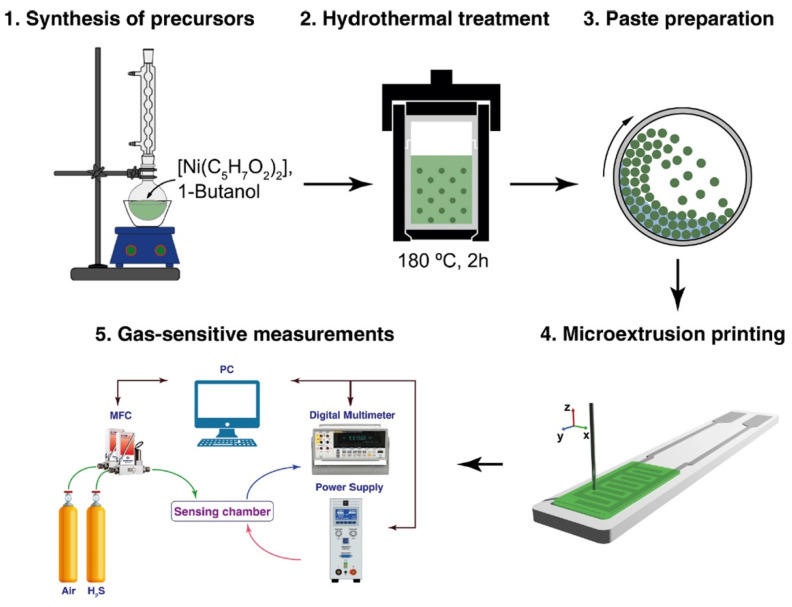
Scheme of hydrothermal synthesis of anisotropic NiO nanostructures and preparation of the ink on their basis that are used for the microextrusion printing of oxide receptor layers. Reproduced from ref. [88]. Copyright 2021, Elsevier, Ltd.

These protocols normally enable a microdosing of ink in a specified area of the substrate, which can have a complex shape as shown, for instance, in one of the first works devoted to prototyping gas sensors with such a printing [88]; see Figure 5. In this study, using the hydrolytically active organometallic nickel compound as a precursor, the NiO nanosheets were obtained under hydrothermal conditions and used to prepare a paste-like ink. After applying the coating to the Pt/Al_2_O_3_/Pt chip surface, it has been shown that the layer has a sufficiently high porosity and the average NiO particle size is about 32 nm. In course of chemisensor measurements, it was found that the material exhibits a high sensor response in forward to detection of hydrogen sulfide. In particular, the response to 100 ppm H_2_S for the prepared oxide coating was 9786%.

### 4.5. Pen Plotter Printing

Pen plotter printing started taking a lot of attention recently, which is supported by high publication activity. Usually, pen plotters are employed with a pen utilized to print the pattern. The pen is filled with inks and further installed in a special holder. Plotters move in the X and Y planes and are unable to move in the Z plane, giving a chance to obtain planar layers only. This technology allows one to apply layers of various geometries, and to fabricate the pattern using a special software to enable further multiple reproductions. The quality of the printed layers depends on the geometry of the tip of the pen, ink viscosity, substrate wettability, and application mode with particular speed and resolution [101]. The thickness of the single layer can be reduced down to 400 nm. Applications of the pen plotter printing method include fabrication of sensitive layers for gas sensors [102], electrodes for supercapacitors [101], microfluidics field [103], deposition of electrodes (AgNP and CNT) on paper substrates [104], and biosensors on paper substrate for diagnostics-related applications [105]. Despite its simplicity and the availability of equipment, this method is not often used in the fabrication of gas sensors today [102].

Nevertheless, pen plotter printing is a rather versatile approach, allowing one to use both true solutions of reagents or organometallic precursors and dispersions of nanoparticles in various solvents as inks.

**Figure 6:**
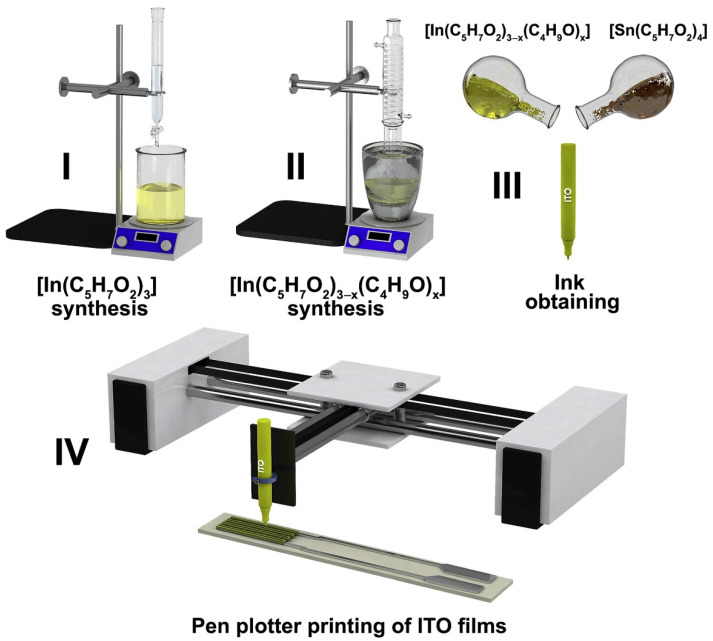
Scheme of the ink obtaining and pen plotter printing of ITO films. Reproduced from ref. [102]. Copyright 2021, Elsevier, Ltd.

Recently, this technique was applied to design thin-film receptor layers using alcohol solutions of hydrolytically active heteroligand complexes—indium and tin acoxoacetylacetonates in order to obtain ITO films (Figure 6) [102] or similar coordination compounds of cobalt, in the case of Co_3_O_4_ films’ application [101], as inks. The gas sensor based on the printed ITO thin film demonstrated a high sensitivity to CO, down to 100 ppb concentration, and the sensor based on the printed Co_3_O_4_ thin film as a receptor component displayed a sensitivity to CO, down to 4 ppm, and NO_2_, down to 100 ppm, depending on the working temperature [101]. Thus, pen plotter printing is a rather convenient and affordable method for producing gas sensors based on thin-film structures of different chemical composition, including ones under a complex geometry.

### 4.6. Microplotter Printing

Microplotter printing technology is a fairly new method that has not been studied much yet. In the process of printing, microplotter moves the plotting unit, i.e., piezo element and glass capillary dispenser [106]. The piezoelectric element, in turn, helps to control the transfer rate of liquid to the surface of target; the gentle pumping of fluid to the surface occurs when the glass capillary dispenser is driven at frequencies in the range of 400–700 kHz. This method provides a high level of accuracy, both in the dosing of functional ink and the plotting, as well as enables a continuous printing, which yields a chance to obtain continuous lines and shapes. Frequently, the Sonoplot Microplotter II is employed for printing [106,107,108,109,110] which operates with inks at viscosity of 0–450 cP; the drop volume starts usually from 0.6 pL, and the resolution is about 5 µm.

**Figure 7:**
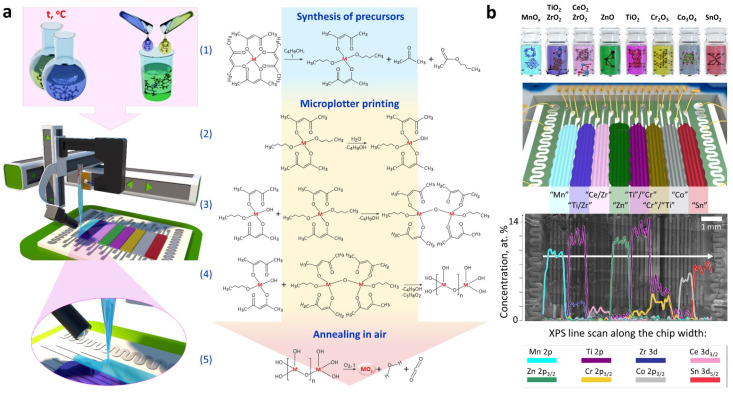
On-chip multioxide combinatorial library printed by microplotter: (**a**) Schematic illustration of the fabrication process including the cartoon of the experimental printing setup with a microplotter and chemical routes for heteroligand precursor synthesis (1), their hydrolysis (2), condensation (3), polycondensation (4), and oxide crystallization (5); (**b**) Chip prototype fabricated with oxides of the list drawn at the first line: the animation drawing and SEM image; the concentration of the different metal elements along the chip surface detected by XPS scanning in the direction indicated by a white arrow; the signals related to O 1s stemming from the substrate/printed layers and Si 2p from the substrate are not depicted here. Reproduced from ref. [107]. Copyright 2020, American Chemical Society.

Such a device, as the aforementioned, makes it easy to replace an ink without replacing a cartridge. The characteristics of the plotted layers depend mainly on the ink properties. For example, utilization of dispersions requires a control of the particle size and depends on the solvent, transition kinetics, and aging. Normally, major attention is paid to the viscosity and surface tension of inks. Glycerin, ethylene glycol, polyvinyl alcohol, and sodium carboxymethylcellulose are commonly added to adjust the ink’s viscosity. Water and ethylene glycol are often used to prepare inks based on particle dispersions. The thickness of a layer might be less than 100 nm [107]. The use of microplotter printing is possible in such fields as gas [111] and optical [110] sensors, biosensors [108], solid electrolytes [106], photocatalysts [109], and microlenses [112]. The technique is applied for metallization of the surface [113], as well as in flexible electronics [114,115], stretchable thin-film transistors and integrated logic circuits [116], RGB displays [117,118], and composite electrodes [119]. Nowadays, this method is not frequently used to design gas sensors. However, it is still promising because of a number of advantages [111]. For example, due to the possibility of a fast cleanup of the capillary dispenser from functional inks before filling it with inks of different composition, the microplotter printing is a very effective way to fabricate multisensor arrays such as combinatorial libraries of receptor components with different chemical composition over a single chip (Figure 7). For instance, using solutions of organometallic compounds as functional inks for microplotter printing, an array of 8 receptor semiconductor layers differing both in chemical composition (Mn_3_O_4_, TiO_2_/ZrO_2_, CeO_2_/ZrO_2_, ZnO, TiO_2_, Cr_2_O_3_, Co_3_O_4_, SnO_2_) and a conductivity type was formed on the surface of a multi-electrode chip [107]. This allowed the device to work in the mode of recognition of various analytes. At the same time, a sufficiently high resolution of printing makes it possible to implement the miniaturization of such devices. In addition to solutions, inks composed of dispersions of nanoparticles in various solvents can also be employed in these protocols. In particular, using a paste based on ZnO particles decorated with Pt nanoparticles, thick-film composites were obtained [120]. It was found that such a modification of zinc oxide particles with the noble metal reduces the sensitivity to NO_2_ and CO but enhances it to benzene and hydrogen, which is explained by chemical and electronic sensibilization.

### 4.7. Screen Printing

**Figure 8:**
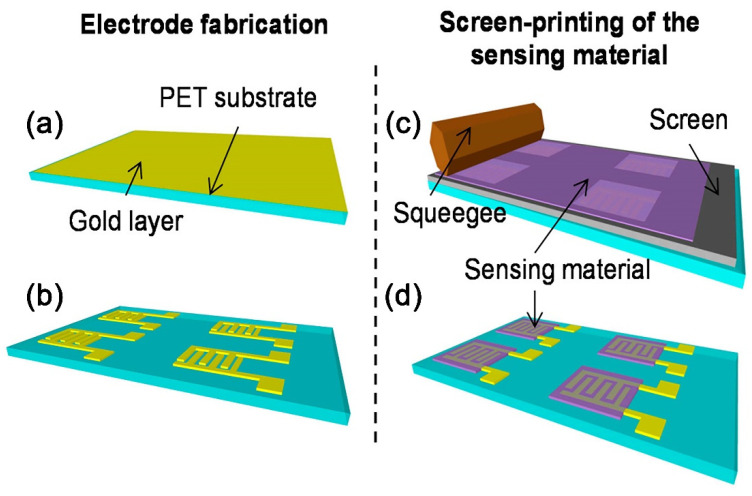
The fabrication of humidity sensors by screen printing: (**a**) deposition of the Au layer on PET (Poly-Ethylene Terephthalate) substrate; (**b**) laser ablation of the Au layer; (**c**) screen printing of the TiO_2_ nanoparticles; (**d**) sensors after screen printing. Reproduced from ref. [124]. Copyright 2017, MDPI.

The screen printing method envelops two technique types: flat-bed and rotary [121]. In the case of flat-bed printing, the ink is pressed through a flat stencil to print the pattern on the substrate. The blade moves along the surface of the stencil and removes the ink residuals. The rotary technique is realized by pressing the inks through a cylinder made of polyester and perforated metal to transfer inks to the substrate. The cylinder rotates at the same speed as the substrate, and the inks are constantly pressed against the substrate using a fixed blade. Comparing these two techniques, one can say that the rotary variant of screen printing allows us to achieve a much higher printing speed when compared to the flat-bed one. In general, screen printing requires a control of the concentration of functional ink, the resolution of the stencil, the proportion of ink transferred to the substrate, the characteristics of the substrate, and the ink viscosity. Usually, the viscosity of ink applied in this method is quite high. Commercially available inks have the highest content of solid particles, about 60–70%, which, in addition to a large particle size distribution (from hundreds of nanometers to tens of microns), leads to high ink viscosity, from 10^2^ to 10^6^ mPa·s [122,123]. Due to its simplicity, this method is one of the most popular one for a production of a wide range of gas-sensing materials. For example, screen printing is used in order to apply appropriate semiconductor coatings over the surface of flexible substrates. In particular, using this approach, Dubourg et al. [124] produced miniaturized TiO_2_ chemosensor coatings on PET (Poly-Ethylene Terephthalate) substrate (Figure 8). A paste based on cellulose and TiO_2_ nanoparticles was used as an ink. As a result, it was shown that the fabricated sensors had an excellent mechanical stability, relatively fast response/recovery times, and high reproducibility for humidity measurements, in the range from 5% to 70% of rel. humidity at room temperature.

Screen printing has also been used to develop gas sensors versus formaldehyde detection [125], where a thick SnO_2_ film was applied as a sensitive material. To increase the selectivity of the material to formaldehyde, the zeolite coatings of different thicknesses were applied on top of the surface of the tin dioxide layer to serve primarily as filters for analyte adsorption. It is worth noting that screen printing is also applied to design electrochemical sensors [126,127,128], flexible [129] and wearable electronics [130], solar cells [131], thermocouples [132], electrodes [133], displays, and secondary power sources.

### 4.8. Gravure Printing

Gravure printing represents a promising high-performance roll-to-roll method characterized by a high rate of printing of functional inks over large areas at low costs [134]. The method protocols normally include three operational stages: filling of cells of the template with inks, ink surplus removal, and ink transfer to the substrate. First, the inks are delivered to an engraved printing cylinder. During the printing, the rotating cylinder transfers the inks to the substrate. Thus, the printing cylinder is partially placed in a container with inks that ensures filling engraved cells of the gravure cylinder with them. A printed pattern is a replica of a pattern engraved on the cylinder. Surplus of inks is collected by doctor blade placed in a close contact with the rotating cylinder. Inks are transferred from the surface of the cylinder to the substrate with the help of an impression roller. The protocols usually employ low-viscosity inks [135]. The quality of the printed layers depends on (1) physical properties of the inks as viscosity, rheological characteristics, surface tension, rate of solvent evaporation [136], (2) substrate (surface tension, porosity, roughness), and (3) the printing process (geometry and density of engravings, printing speed). This technique enables printing layers with thicknesses down to 20 nm [137], just adjusting the contact force and depth of engravings on various flexible substrates [138]. In the frame of this method, the printing rate might be increased, up to 15 m/s, without severe losses in the resolution of the printed pattern [139].

**Figure 9:**
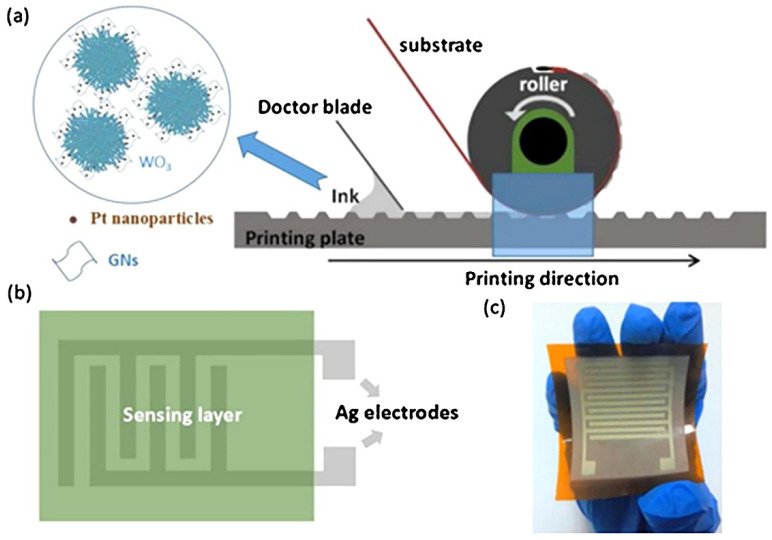
(**a**) Schematic representation of the gravure printing process; (**b**) Schematic of the printed sensor; (**c**) Photo of a printed WO_3_/Pt-GNs sensor on a PI substrate. Reproduced from ref. [145]. Copyright 2018, Elsevier, Ltd.

Currently, the gravure printing applications include electronic [134,138,139,140,141,142,143,144] and power [136] industries. In the field of gas sensors, this method suits fabricating the sensors over flexible substrates. For instance, Chen et al. [145] applied the technique to deposit a composite receptor layer of WO_3_/reduced graphene oxide (GO) nanosheets decorated with Pt nanoparticles (Figure 9). In this case, the dependence of the material’s sensor properties on the size of the graphene structures was shown. In particular, the introduction of small-sized nanosheets of reduced GO into the active layer provided an open surface state of the tungsten oxide particles and, consequently, a quite selective sensor response to low acetone concentrations combined with a low gas response/recovery time.

Using gravure printing, ZnO nanoparticle seed layers were also deposited on the surface of various types of substrates, including flexible ones, for a subsequent growth by chemical bath deposition of structures based on zinc oxide nanowires (NWs) to be promising for use as a piezoelectric sensor component [146]. Thus, this printing can be easily applied as a technological element in frames of multistage processes of manufacturing various functional nanomaterials.

### 4.9. Flexographic Printing

**Figure 10:**
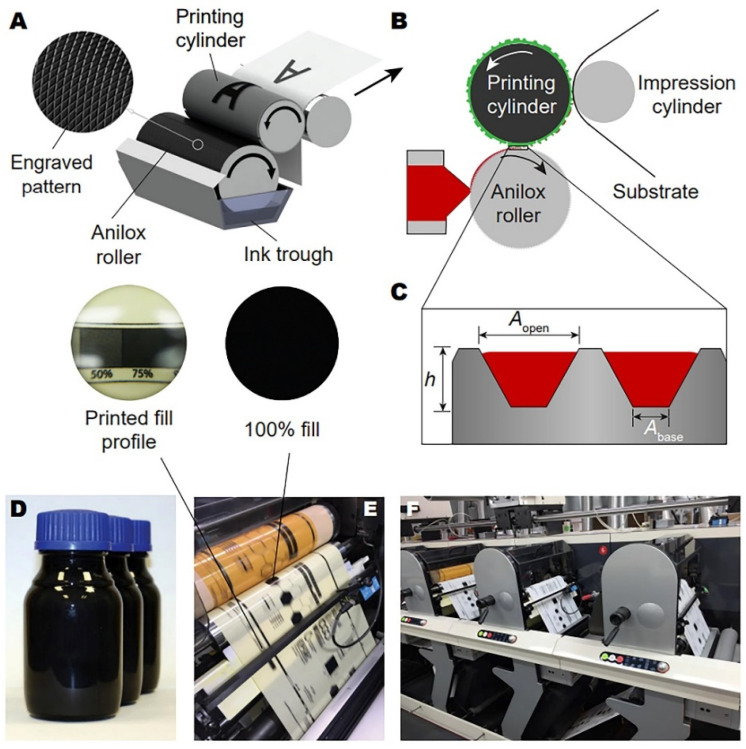
Flexographic printing: (**A**–**C**) Schematic figures showing flexographic printing principles; (**D**) Photograph of graphene/carbon flexographic ink. Printing trials of the ink shown in (**D**) on (**E**) PET, and (**F**) paper substrates using a commercial graphics printing press. Inset: zoomed-in photographs of the printed fill profiles. Reproduced from ref. [21]. Copyright 2018, Royal Society of Chemistry.

In flexographic printing, inks from a container are transferred to an anilox roller to fill the engraved cells as basically shown in Figure 10 [21]. Then, the inks go from the roller to photopolymeric flexographic master mold placed at a plate cylinder. At this stage, the inks are subjected to both shear and tensile stresses. Further, the inks are carried from the mold to the substrate on the printing cylinder for drying to complete the printing process. Flexible printing molds with relief patterns, normally made of resin or photopolymers, along with liquid fast-drying inks of low viscosity, are usually employed in this method [147].

The primary advantage of flexographic printing adheres to a broad scope of suitable substrates [148,149,150], and a wide range of viscosity of inks, 50–500 cP, which could be utilized for printing. Still, the possible appearance of so-called ‘halo effects’ has been identified and related to the accumulation of surplus of inks at ages [151]. A high-performing flexographic printing assumes control of viscosity, surface tension, and elasticity of inks [152] that also yields printing resolution [153,154]. The flexographic printing is a popular industrial method. In addition to the application for printing various packages and labels, flexographic printing can be applied to the fabrication of gas sensors and biosensors [155]. For instance, flexographic printing allows designing chemosensory structures having various compositions on flexible substrates. In particular, Lloyd et al. [156] used this method to modify silicon and polyimide substrates by depositing ZnO nanoparticle seed films on their surface followed by growing vertically oriented arrays of ZnO NWs in hydrothermal conditions. The use of receptor components containing anisotropic, also at one-dimensional architecture, nanostructures can help to significantly promote the efficiency of gas sensors. In this case, the fabricated materials showed a high oxygen response. Due to technological peculiarities, the flexographic printing method is currently not widespread in the fabrication of gas sensors. Nevertheless, this approach can be considered promising in terms of mass production of corresponding devices with a sufficiently high spatial resolution, including applications in areas such as electronics [157,158,159], power industry [149,160], photonics [161], and medicine [151].

### 4.10. Laser-Induced Forward Transfer (LIFT)

**Figure 11:**
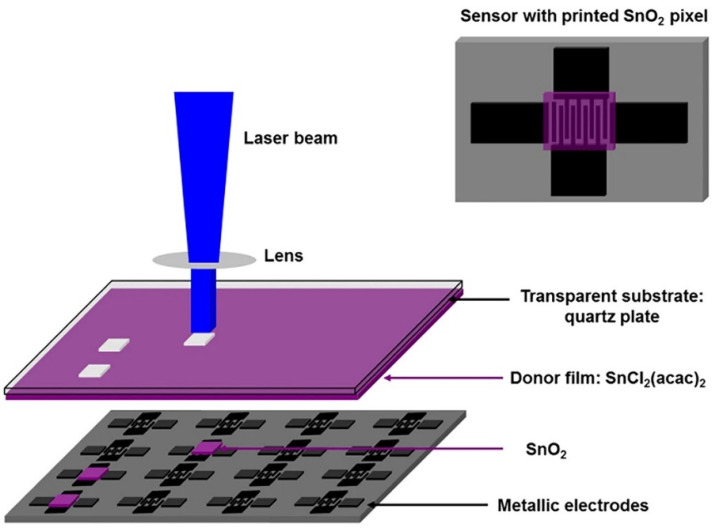
Scheme of the reactive laser-induced forward transfer process. Reproduced from Ref. [164]. Copyright 2016, Nature Publishing Group.

The laser transfer method has been known since the late 60s, but it began to develop rather intensively after 1986 when the LIFT term was introduced [162]. This method includes many different “sub-methods”, for example, laser-induced backward transfer (LIBT), matrix-assisted pulsed laser evaporation (MAPLE), blister-actuated LIFT (BA-LIFT), biological laser printing (BioLP), absorbing film assisted-LIFT (AFA-LIFT), and others. All these protocols employ a pulsed laser to transfer a material from the pristine (donor) substrate to a given (acceptor/receiver) substrate. The donor substrate is usually a transparent material, for example, quartz or glass, which are covered with a thin film of the target material to be transferred. After the laser pulse irradiates the interface between the donor substrate and the transferred material, the material binds to the receiving substrate. The factors which define characteristics of the printed layers are the uniformity of the donor coating (the most homogeneous one are normally required), the pristine substrate, and a viscosity of inks in the case of their transfer [123]. The LIFT method has a number of advantages, which include a high spatial resolution, the ability to transfer materials in both liquid and solid phases, and the opportunity to utilize functional inks with a wide-ranged viscosity, 1–300 mPa·s. This method was frequently reported to design chemical sensors. As examples, we may note the matrix-assisted pulsed laser evacuation (MAPLE) and MAPLE direct-write (MAPLE DW) protocols which are delivered in Ref. [163]. Both of these protocols have been successfully applied to fabricate thin films and structures from a number of organic materials. Further, the LIFT approach was employed recently to deposit the receptor materials, of SnO_2_ and Pd:SnO_2_, which demonstrated a higher sensing response to ethanol and methane (Figure 11) compared to commercial counterparts [164]. The authors chose SnCl_2_(acac)_2_ and Pd(acac)_2_ coordination compounds as precursors to form donor materials, as thin films on the surface of a quartz substrate, by a spin coating. The LIFT method was also shown to allow designing of reproducible sensors based on SWCNT:SnO_2_ hybrid nanocomposites [165]. In this case, the obtained material was characterized by a low ca. detection limit to ammonia detection at the level of 0.59 ppb under room temperature conditions. Thus, the considered approach makes it possible to form high-resolution miniaturized planar nanostructures which exhibit competitive functional properties.

At the same time, this technique is rather complicated to be dependent on the prefabrication of donor materials, whose characteristics largely ensure the efficiency of the deposition process and the output properties of the target materials. According to literature, LIFT can be used not only in the field of gas sensors [162], but also is expected to help in designing RFID tags, various biosensors, alternative energy devices, e.g., solar cells [166], and wearable electronics [167,168,169,170,171,172]. The prospects of this technology were recently discussed in detail by Delaporte and Alloncle [173].

### 4.11. Dip-Pen Nanolithography (DPN)

**Figure 12:**
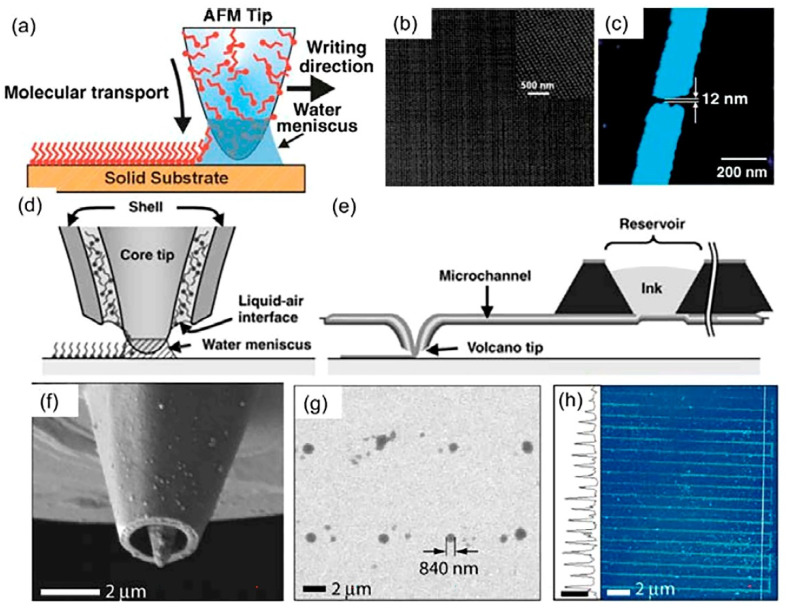
(**a**) Schematic representation of the dip pen nanolithography (DPN) process. A water meniscus forms in area between tip and substrate which facilitates a molecular transport from the tip to the target substrate; (**b**) Tapping mode AFM images of 60 nm Au nanodots deposited by DPN and subsequent etching; (**c**) 12 nm Au nanogap fabricated by DPN and subsequent etching; (**d**) Schematic representation of fountain pen nanolithography. The ink is dispensed through the hollow tip to the substrate; (**e**) Schematic representation of the nanofountain pen probe structure. A micro reservoir for storing inks is connected to the volcano tip through a microfluidic channel; (**f**) SEM image of the volcano tip; (**g**) SEM image of a 2 × 4 array of anti-BSA IgG dots patterned on a BSA substrate (46% RH) by fountain pen; (**h**) Tapping-mode AFM image and height profile of parallel lines of biotin-BSA patterned on 6-Mercaptohexanoic acid (MHA) at a translation rate of 80 μm/s (50% RH, height scale bar in profile is 20 nm). Reproduced from Ref. [174]. Copyright 2017, MDPI.

DPN employs an atomic microscope probe, i.e., cantilever, dipped, or vapor coated into inks as a writing unit. Application of inks at the surface of the substrate is favored by the appearance of a meniscus formed either by condensation of air moisture or by the ink itself. The meniscus connects the tip of the cantilever and the surface of the substrate as shown in Figure 12 [174]. The process can be divided into three stages: (1) dissolution of molecules deposited on the cantilever/tip in the meniscus; (2) diffusion of molecules from the tip to the substrate through the meniscus; (3) assembly of molecules on the substrate by using various methods of processing, for example, by thermal one, or chemical reaction [175]. The factors influencing characteristics of the printed layers include chemical and physical characteristics of the ink, surface tension, tip structure, Laplace pressure on the meniscus, exposure time, contact force, lifting speed, and ambient humidity [176]. There are some reports delivering an evaluation of temperature influence on dimensions of obtained layers [177].

In these protocols, an important role is attributed to the inks, which are distinguished in two groups: diffusive and liquid ones. Diffusive inks are usually based on water-soluble molecules that can create self-assembled monolayers (SAM), while liquid ones are viscoelastic liquids whose surface tension is discussed to play a greater role for transporting target molecules, and not meniscus [175]. Dip-pen nanolithography is shown to be applied to the formation of SWCNTs layers for solar cells [178], biomedical diagnostics, nanoelectronics, patterning of biomolecules [179,180], polymers [175], and catalysts [181]. This method is not frequently employed to design gas sensors; nevertheless, there are some examples in this field, too. For instance, Lu et al. [182] used DPN to fabricate a low-temperature gas sensor to NO detection, where NWs made of a conducting polymer (poly(3,4-ethylenedioxythiophene, PEDOT) with a diameter of 300 nm were printed in the gap between interdigitated microelectrodes to serve as the receptor material. The DPN method was also used to prototype a gas sensor sensitive to CO_2_ [183]. In this case, the doped polypyrrole was used as the receptor material prepared as 5 wt.% solution in water. The material was applied using Si_3_N_4_ contact cantilever with a rounding radius of about 20 nm. Before the material was deposited on the substrate, the cantilever was immersed in the ink for a minute and then purged with N_2_. As a result, a miniature gas sensor which had a quite low response time, of 9 s, versus 18 ppm of CO_2_ was made. Thus, the dip-pen nanolithography method has all the options to allow developing chemisensor structures with a high resolution which is still limited by a necessity to employ a sophisticated equipment.

### 4.12. Nano-Imprinting Lithography (NIL)

Nano-imprinting lithography defines a group of soft lithography methods, which requires a rigid stamp, and a polymer layer applied to a substrate. The particular protocol depends on the type of NIL method which are distinguished as thermal NIL, UV NIL, two-photon nanolithography [184,185], laser-assisted direct imprint (LADI) [186,187], nano-electrode lithography, and soft nanolithography [188]. In the case of the thermal NIL, the polymer layer and a stamp are heated above the glass transition temperature, then the stamp is evenly pressed against the polymer layer to be further used for printing. UV NIL uses UV for making micro- and nanopatterns. The UV curable polymer is applied at the substrate by a spin-coating method. Then, the stamp is pressed against the substrate with a polymer layer and the polymer is cured by UV illumination. In two-photon nanolithography, the laser is focused through optics placed in a container with photosensitive polymer. Further, fabrication is carried out using a layer-by-layer process. This method enables the printing of elements with dimensions even less than 10 nm. The advantages of these protocols include high resolution, high speed compared to other printing methods, easily scalable, simple, and a flexible process. Among the disadvantages, we may note the need to control the force: if the applied force is too low, the pattern on the surface will not be fully reproduced, while excessive forces can damage the stamp. In addition, the stamp can wear out quickly due to the high temperature and force applied [189].

**Figure 13:**
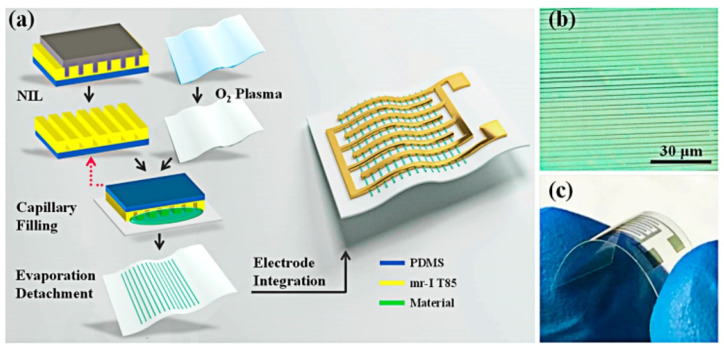
(**a**) Schematic illustration of the preparation process of flexible NW-based sensors under NIL protocol; (**b**) Optical microscope image of the highly aligned PEDOT:PSS NWs; (**c**) Picture of the integrated flexible device. Reproduced from Ref. [188]. Copyright 2019, American Chemical Society.

This group of methods is favored to design LEDs [189] and gas sensors [188]. In particular, it was used to fabricate a low-power consumption ammonia sensor [188] as shown in Figure 13. Densely ordered and oriented NWs based on PEDOT:PSS, ZnO, GO, and In(NO_3_)_3_ up to 100 nm thick, were deposited on the surface of a flexible PET substrate. The sensor was shown to retain a mechanical flexibility and durability after 1200 bending cycles without degrading the performance. Consequently, the sensor demonstrated a high selectivity and sensitivity to NH_3_ with a power consumption of up to 3 μW. A similar protocol was also applied to fabricate a flexible hydrogen sensor with a response time at 50 ppm of H_2_ equal to 57 s), where 1D Pd structures were formed as a high-resolution sensing component [190]. The obtained materials were similar in their microstructural characteristics to those discussed in the Section 4.11. Altogether, NIL protocols make it possible to design miniature highly-efficient gas-sensing materials at a high resolution. However, their application is limited by the necessity of template fabrication which complicates the widespread use of this technology.

### 4.13. Microcontact Printing (µCP)

The µCP is a soft lithographic technique that enables the printing of patterns at flat and curved substrates [191]. This method is often utilized for the particular fabrication of self-assembled monolayers (SAM) [192]. As a substrate, one can use polymers, Pd, Ag, Au, while elastomers, e.g., PDMS, are used to prepare a stamp [191,193,194,195]. Inks are applied to the substrate via a chemisorption and/or physical adsorption.

**Figure 14:**
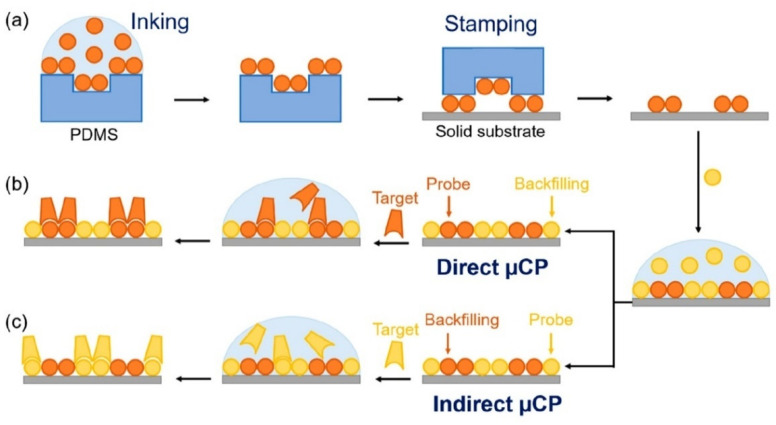
Schematic illustration of the fabrication and biorecognition processes, including (**a**) inking and stamping, (**b**) standard microcontact printing (μCP), and (**c**) indirect μCP. Note that in standard μCP, probes are patterned by stamping, whereas in indirect μCP, backfilling agents are stamped first and then the probes are physisorbed on the gaps just by incubation. Reproduced from ref. [200]. Copyright 2018, MDPI.

The layer characteristics depend on the molecular weight of the transferred compounds, the concentration of the solution, and the pH of the solution. The higher the molecular weight, concentration, and pH values of the solution, the greater speed of µCP [193]. There are several variations of the µCP method to include high-speed µCP, submerged µCP, microdisplacement µCP, and positive µCP [196]. For example, high-speed µCP implies that reducing the contact time of the stamp with the substrate to milliseconds improves the uniform and reproducible printing of the monolayer. This method can be easily scaled without a loss in the printing quality [197]. So far, microcontact printing has primary applications in fabrication of electrochemical devices [198], electronics [197], and biotechnologies [199,200,201]; see Figure 14. These protocols have also been tested to design gas sensors. In particular, a material based on WO_3_ nanorods sensitive to H_2_ was fabricated in a study [202]. Such one-dimensional WO_3_ nanostructures were printed as a self-organized array on muscovite-type mica using the vapor deposition process. Next, accurately aligned, individual, Pt μ-wires were applied to their surface in order to measure their electrical properties. The resulting material allows one to detect hydrogen at concentrations down to 50 ppm. µCP protocols were shown to assist in the fabrication of C_3_H_8_, CO, and NO sensors [203]. In this case, hierarchically organized thin films of ZnO nanosheets, obtained on local areas of the Al/SiO_2_/Si substrate by chemical deposition upon reaction between zinc nitrate and urea at elevated temperature, were used as the receptor material. The optimum operating temperatures were 350 °C, 400 °C and 200 °C for C_3_H_8_ (5000 ppm), CO (250 ppm), and NO (1000 ppm) detection, respectively. Therefore, microcontact printing makes it possible to produce receptor components of gas sensors with high resolution, although, similar to the nano-imprinting lithography method, there are limitations in its wide applicability due to the requirement for template fabrication to suit each specific application.

## 5. Features of Functional Inks Used in the Formation of Receptor Components for Gas Sensors by Various Printing Methods

As can be seen from the previous section, the formation of gas-sensor receptor layers, which depends on the features of the printing method used, differs not only in the process speed, spatial resolution, and thickness of the obtained film, but also in the corresponding requirements for the inks (rheological characteristics, composition, etc.); see Table A1 (Appendix A). So far, ink-jet printing, pen plotter printing, microplotter printing, dip-pen nanolithography, and microcontact printing typically employ low-viscosity inks. They can be either true solutions of reagents [203] or organometallic precursors [101,102,107], or have the form of dispersed systems, such as colloidal solutions or stable suspensions.

In the case of using solution ink, a chemical transformation can be initiated on the substrate surface with the formation of target products, whose microstructure is determined by the conditions of this process. In some cases, additional heat treatment of the coating is required in order to crystallize the material. These methods are mainly used to form thin-film receptor materials and provide a sufficiently high print resolution. In such methods as laser-induced forward transfer, gravure printing, flexographic printing, and aerosol jet printing, the ink viscosity can vary over a wider range, but in most cases, the medium-viscosity inks are required to successfully apply the coatings. In some cases, an additional heat treatment of the material is also required posterior to the film and is applied by these methods. More viscous inks are used in 3D printing, screen printing, and microextrusion printing to form functional coatings. As a rule, they appeared as pastes containing disperse phase particles, organic binder, and solvent [124]. After the coating deposition, a heat treatment is required to remove the solvent and binder. As a result, the coatings obtained by these methods in most cases are quite porous, which increases their sorption activity and, accordingly, the sensitivity when detecting various analytes. Thus, depending on the rheological properties of the ink, its composition, method, and printing conditions, thin- and thick-film materials with different porosity and spatial resolution from 2 nm to 1 mm can be obtained, which directly determines their electrophysical and chemisensory characteristics.

## 6. Conclusions

The literature review showed that printing technologies are nowadays an integral element of contemporary materials science applied to development of low-cost gas sensors and multisensor arrays for many applications including a development of lab-on- chips. These protocols ensure automating of technological processes, a reproducibility of microstructural and functional characteristics with a reduced time necessary for the receptor material deposition over substrates. At the same time, using an accurate positioning system improves significantly the targeting of the substance, while the dosing setups allow to ensure a high control over the volume of discretely or continuously applied inks. The printing technologies enable forming planar receptor structures, even under a complex geometry, at various thicknesses and porosity with the required spatial resolution to be in nanometer micrometer ranges. Some methods, as dip-pen nanolithography, nano-imprinting lithography, and microcontact printing, are more suitable for discrete miniature devices with unique characteristics owing to the labor-intensive and multi-step procedures, while other ones, as ink-jet printing, aerosol jet printing or microextrusion printing, can be used quite easily in scaling the procedures to design gas sensors, including a rapid tuning of their geometric parameters without a necessity to prepare appropriate stencils and masks in advance. A number of methods, such as microplotter printing, due to the possibility of fast cleanup or replacement of dispensers, are extremely convenient for quick printing receptor layers with sufficiently high resolution to meet the requirements of multisensor arrays, where a number of receptor layers of different chemical composition are placed over the chip crystal surface. It should be noted that the choice of the most suitable printing technology and the properties of the deposited chemisensor material also strongly depend on the rheological properties of the ink employed, whose viscosity can vary by orders of magnitude. The review highlights some of the most accessible printing methods (pen plotter printing, microplotter printing, and microextrusion printing), which have only recently become available to the broader scientific and technological community for the development of gas sensors.

## Data Availability

Not applicable.

## Appendix A

**Table A1:** The key characteristics of the printing technologies employed in gas sensor fabrication.

Method	Speed; Resolution; Thickness	Ink Viscos-ity, mPa·s	Material	Detected Analyte	Advantages	Disadvantages	Refs.
Ink-jet printing	1–500 m/min; 0.4–50 µm; 0.015–20 um	1–100	Au, Pt, NP, Pd, Graphene oxide, CNT, OFETs, SnO_2_-rGO, SEBS, α-Fe_2_O_3_/rGO, TiO_2_–10%ZrO_2_, SnO_2_	O_2_, S^2−^, K^+^, H_2_O_2_, Cl_2_, Glucose, NH_3_, NO_2_, CO_2_, H_2_S, Ethanol, Formalde-hyde, CO, Pentane, Heptane, Acetone, H_2_	Possibility of programming the coating process; High speed of printing; Biocompatibility; Simple control over the thickness of the coating; Low risk of contamination; Low ink consumption; Possibility of creating the multisensory coating.	The number of cartridges is limited; Software limitations; Clogging of printhead and nozzles.	[16,18,25,30,49,50,54,55,56,204,205,206,207,208,209,210,211,212,213]
Aerosol jet printing	0.1–200 mm/s; 10 µm; 30–1000 nm	1–1000	Pt-SWNCTs, Graphene, ZnO, SnO_2_, Pt/SnO_2_, TiO_2_, SrTi _0_._7_Fe_0_._3_O_3-x_, Pd/SnO_2_, Pd/Al_2_O_3_, Y_2_O_3_-ZrO_2_	H_2_, NH_3_, CO, Ethanol, O_2_, Propane, Methane, NO, NO_2_	High resolution; Convenient control over the printing; High efficiency; A wide range of ink viscosity is available.	Overspray; Clogging of the nozzle.	[15,18,30,57,58,59,60,63,64,65,66,68,69,70,214,215,216,217]
3D printing	50–600 mm/min; 50–250 µm; 250–1000 um	10^3^–10^9^	CuO, CuO/Cu_2_O/Cu—Fe_2_O_3_/Fe, PBS/graphene	NH_3_, Acetone, Methanol, Hexane, Toluene, H_2_O, diethyl ether, 1,4-dioxane, dimethyl carbonate	A wide range of materials is suitable for printing; The method has many types; Possibility to quickly changing applied structures by software and easy to control; Relatively cheap printers; Low material consumption.	Method is too slow for using on a large scale.	[71,74,75,80,82,83,84,218,219,220]
Microextru-sion printing	1–10,000 µm/s; 5–1000 µm; 5–500 um	1–10^8^	NiO	H_2_S	A wide range of materials is suitable for printing; Cheapness of the method; Low ink consumption; Possibility to quickly changing applied structures by software and easy to control.	Method is too slow for using on a large scale.	[86,87,88,89,90,91,93,94,95,96,97,98,99]
Pen plotter printing	50–5000 mm/min; 50 µm; 20–400 nm	4.25–40	Co_3_O_4_, ITO	H_2_, Methane, CO, NO_2_, CO_2_, NH_3_	Continual supply of material; Absence of strict requirements to the rheology of the ink; There are no limitations on the size of substrate; Possibility of using different types of substrates (including flexible) and its size; Possibility to quickly changing applied structures by software; Very cheap method.	Relatively high roughness; Low reproducibility; Method is hard to adapt for a large scale.	[7,101,102,103,104,105,221]
Microplot-ter printing	1–2 mm/s; 5 µm; 75–200 nm	<450	ZnO/Pt, Mn_3_O_4_, TiO_2_/ZrO_2_, CeO_2_/ZrO_2_, ZnO, TiO_2_, Cr_2_O_3_, Co_3_O_4_, SnO_2_	CO, NH_3_, H_2_, NO_2_, Benzene, Ethanol, Methanol, Isopropanol, n-butanol	Inexpensive method; Relatively simple method; Possibility to quickly changing applied structures by software and easy to control.	Relatively slow method; Method is hard to adapt for a large scale.	[107,112,114,116,120]
Screen printing	5–150 m/min; 50–100 µm; 3–100 um	500–50,000	SnO_2_, CdS-SnO_2_, ZnO, Cd-ZnO, CeO_2_, In_2_O_3_, InSnO_x_, ZnO-SnO_2_, TiO_2_/GO	Humidity, Toluene, Ethanol, Methanol, LPG, Acetone, CO, CNG, Hydroxyl-amine	The method is well suited for thick films; A reliable method; Low cost; Low ink consumption.	The method not suited for thin films; High roughness of coatings; Low resolution.	[16,18,25,30,123,124,125,129,132,222,223,224,225,226,227,228,229]
Gravure printing	6–1000 m/min; 0.1–75 µm; 0.1–5 um	1–1000	OFETs, PANI, WO_3_-PEDOT:PSS, WO_3_, pHEMA, Ag-S-RGO, WO_3_/Pt-decorated rGO	Acetone, NH_3_, NO2, NO, H_2_, Humidity, CO	High resolution; Low ink consumption; Possibility of using different types of substrates; The method is well scalable.	Defects may form during the printing; Expensive cylinders for printing.	[9,16,18,25,30,121,136,137,141,142,145,230,231,232,233,234,235,236,237]
Flexogra-phic printing	6–300 m/min; 50–200 µm; 5–3000 nm	20–2000	ZnO	O_2_	Fully automated method; High efficiency; Possibility of using different types of substrates (including flexible); Cheap printing plates.	It is necessary to create a printing plate for a new printing scheme.	[18,21,25,30,121,149,151,152,153,155,156,158,159,160]
Laser-induced forward transfer (LIFT)	1–10 m/s; <1 µm; 10–1500 nm	1–10^2^	CNF, SnO_2_, Pd:SnO_2_	Humidity, Nitrogen dioxide, Ethanol, Methanol, Methane	A wide range of materials can be used for printing; Solid materials can be used for printing; Accurate control over printing; A wide range of ink viscosity can be used, including pastes and dispersions with a large particle size; Mostly porous structures with a large surface area are obtained; Low ink consumption.	High cost; Complex equipment; Fuzzy edges of coatings.	[15,123,162,163,164,165,167,168,169,172,173,238,239]
Dip-pen nanolitho-graphy (DPN)	0.25–1 µm/s; <1 µm; >5 nm	27–45	Doped polypyrrole, PEDOT	CO_2_, NO	A wide range of materials can be used for printing; Accurate control over printing; Resolution can be controlled by replacing the AFM cantilever/tip.	Many parameters should be controlled; It is difficult to create high resolution structures; Small- scale printing; High requirements for equipment.	[15,176,182,183,240,241]
Nano-imprinting lithography (NI)	0.1–60 µm/s; 10–25 nm; 8–100 nm	-	PEDOT:PSS, ZnO, GO, In(NO_3_)_3_, Pd/Au, Pd	NH_3_, H_2_, Humidity, Ethanol	Very high resolution; Relatively fast method to create nanoscale coatings; Easy adaptable method to new structures.	Method is too slow for using in a large scale; Defects may form during the printing; Many parameters should be controlled; Mask should be changed quite often.	[25,175,178,184,188,189,190,242,243,244,245]
Microcon-tact printing (µCP)	1–10 mm/s; 2–100 nm; 50–70 nm	1.9	ZnO, WO_3_	Propane, NO, CO, H_2_	The method is easily scalable; The method is quite reliable and simple.	Problems with defects and impurities.	[107,193,196,197,199,200,202,203]
